# Mechanism of Generation of Therapy Related Leukemia in Response to Anti-Topoisomerase II Agents

**DOI:** 10.3390/ijerph9062075

**Published:** 2012-05-31

**Authors:** Ian G. Cowell, Caroline A. Austin

**Affiliations:** Institute for Cell and Molecular Biosciences, Newcastle University, Newcastle upon Tyne, Tyne and Wear NE2 4HH, UK; Email: caroline.austin@ncl.ac.uk

**Keywords:** topoisomerase II, TOP2, translocation, leukemia, AML, etoposide, mitoxantrone, epirubicin, transcription, carcinogen

## Abstract

Type II DNA topoisomerases have the ability to generate a transient DNA double-strand break through which a second duplex can be passed; an activity essential for DNA decatenation and unknotting. Topoisomerase poisons stabilize the normally transient topoisomerase-induced DSBs and are potent and widely used anticancer drugs. However, their use is associated with therapy-related secondary leukemia, often bearing 11q23 translocations involving the *MLL* gene. We will explain recent discoveries in the fields of topoisomerase biology and transcription that have consequences for our understanding of the etiology of leukemia, especially therapy-related secondary leukemia and describe how these findings may help minimize the occurrence of these neoplasias.

## 1. Introduction

Drugs targeting TOP2 (TOP2 poisons) are important and effective anti-cancer agents, but they are associated with serious side effects including the development of therapy related acute leukemia (t-AL), especially acute myeloid leukemia (t-AML). The incidences of therapy related leukemia are increasing due to better survival rates and the use of more intensive chemotherapy regimens to treat primary cancers and t-AML generally has a poorer prognosis than *de novo* AML [1,2]. t-AML accounts for ~15% of all acute myeloid leukemia cases [1] and occurs after treatment for hematological and non-hematological malignancies. AML is the most common secondary cancer to occur following treatment of childhood acute lymphoblastic leukemia (ALL) for example, where it appears with short latency in a proportion of cases who achieve a first complete remission [3]. t-AML also remains a late complication following treatment of solid tumors including in breast cancer patients treated with epirubicin or mitoxantrone, especially those of a younger age at the time of diagnosis [4,5,6]. Thus t-AML is an important clinical problem. Understanding the mechanisms that cause t-AML may suggest ways to reduce its occurrence. 

**Figure 1:**
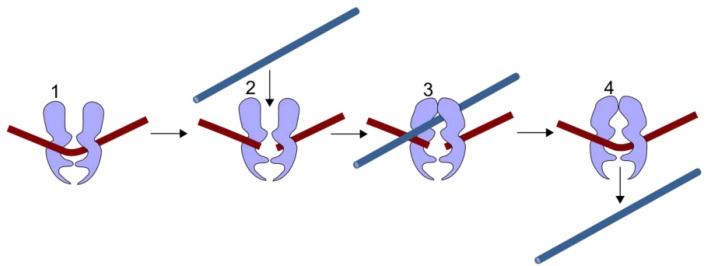
TOP2 mechanism. TOP2 cleaves both strands of a duplex DNA segment (brown, 1–2). A second DNA duplex (blue) passes through the transient enzyme-coupled break (2–3). The first duplex is then re-ligated and the products of the reaction are released from the enzyme (4).

Two classes of anti-cancer agents are associated with t-AML; these are alkylating agents and TOP2 poisons. Both types of agent are cytotoxins widely used in cancer therapy, and achieve their anti-cancer activity by generating DNA damage leading to cell death. Alkylating agents chemically react with DNA to form inter-strand crosslinks and other DNA adducts. In contrast, as described in more detail below and shown in Figure 1, TOP2 poisons interfere with the religation step in the topoisomerase II reaction cycle, leading to the accumulation of DNA double-strand breaks (DSBs). The occurrence of t-AML presumably reflects non-lethal genetic damage induced by these agents in hematopoietic precursor cells, but t-AML cases associated with alkylating agents are biologically and clinically distinct from those associated with TOP2 poisons (see Table 1), suggesting different mechanisms of pathogenesis. 

Monosomy for chromosome 5 or 7 or loss of 5q or 7q chromosome arms are characteristic of alkylating agent-associated t-AML/t-MDS, while karyotypic abnormalities in TOP2 poison associated t-AML are typically balanced chromosome translocations [7] that generate novel fusion genes. Translocations involving the mixed lineage leukemia locus (*MLL*) at 11q23 are the most frequent type of balanced translocation in t-AML [8,9] and acute leukemias with this translocation appear with a short latency period following chemotherapy with TOP2 poisons [7,10]. Other recurrent t-AML translocations include t(15,17)(*PML-RARA*), t(8,21)(*AML-ETO*) and inv(16)(*MYH11-CBFB*) [6]. Chromosome translocations represent a crucial early event in the development of these leukemias and the resulting fusion genes, for example MLL-AF9, are able to transform hematopoietic precursors and induce leukemia in animal models [11]. Cytotoxic drugs such as TOP2 poisons are often administered in complex regimens with other drugs, and this can make it difficult to assess the leukemogenic effect of individual agents. A notable exception to this is the use of mitoxantrone monotherapy to treat multiple sclerosis, where secondary leukemia with *PML-RARA* translocations has been reported as a complication [12,13,14,15,16,17]. How the chromosome translocations observed in t-AL occur and why the same translocations are seen repeatedly has puzzled clinicians and scientists for decades. We will focus on TOP2 poison associated t-AL, starting with the mechanism of action of TOP2 and TOP2 poisons and leading on to recent discoveries in topoisomerase biology that are relevant to the etiology of therapy-related secondary leukemia.

**Table 1:** Typical biological and clinical characteristics of t-AML cases associated with alkylating agents and TOP2 poisons.

	TOP2 poison * associated(* such as etoposide, teniposide mitroxantrone, epirubicin)	Alkylating agent ^$^ associated(^$^ such as cyclophosphamide, melphalan, chlorambucil, and nitrosoureas)
**Latency period**	Short, <2 years	2–8 years
**Chromosome abnormalities**	Recurrent translocations especially involving *MLL* at 11q23 and t(15;17)(*PML-RARA*), t(8,21)(*AML-ETO*)	Deletions involving Chr 5 and 7
**Complex Karyotype**	Rare	Frequent
**Preceded by myelodisplastic syndrome**	Rare	Frequent
**Age association**	Younger	Older

## 2. TOP2 and TOP2 Poisons

**Figure 2:**
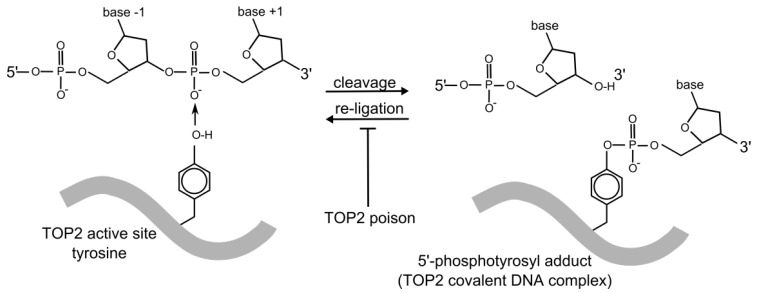
TOP2 DNA cleavage. TOP2-catalyzed strand cleavage involves a reversible, covalent enzyme–DNA adduct formed through the TOP2 active site tyrosine residue. Topoisomerase poisons inhibit the reverse reaction (re-ligation) resulting in stabilization of the 5’ phosphotyrosyl-DNA complex (cleavage complex) which contains a TOP2-linked strand breaks. The TOP2 protein molecule is represented by the thick grey line. Only one TOP2 monomer and one strand of the DNA duplex are shown.

**Figure 3:**
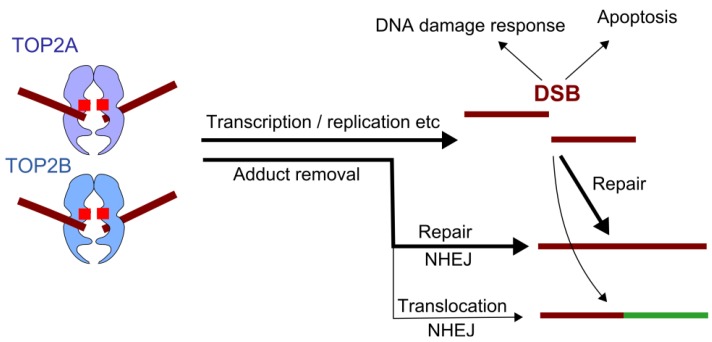
TOP2 Poisons, downstream events. TOP2 poisons inhibit the religation step of the TOP2 reaction cycle, leading to accumulation of covalent TOP2-DNA cleavage complexes. These lesions are cytotoxic and lead to activation of the DNA damage response and potentially apoptosis. Alternatively these lesions are repaired, largely through the non-homologous end-joining pathway. Translocations observed in therapy-related leukemia are presumed to occur as a result of mis-repair, joining two heterologous ends.

DNA topoisomerase II, referred to as TOP2, is essential for cell survival and plays a role in replication, transcription, chromosome condensation and segregation [18,19,20,21,22]. It allows one double-stranded DNA segment to pass through another, thus altering DNA topology. This is achieved by introducing a TOP2-bridged double-strand break into one DNA duplex, where each monomer of the dimeric enzyme remains covalently attached to the ends of the DSB via a 5’-phosphotyrosyl linkage (Figure 1 and Figure 2). A second DNA segment is then passed through the enzyme-bridged DNA gate, and the break is re-ligated. The enzyme-bridged gate is normally a short lived intermediate, but TOP2 poisons inhibit the religation step, resulting in the formation of an unusual type of DSB called a cleavage complex, in which the topoisomerase protein remains covalently coupled to the DNA (Figure 2 and Figure 3). These breaks are cytotoxic, hence the utility of TOP2 in cancer therapy. Unfortunately, TOP2 poisons also have genotoxic side effects, including the formation of leukemogenic chromosome translocations. Humans and other vertebrates possess two type II topoisomerase paralogs, TOP2A and TOP2B, encoded by genes on chromosomes 17 and 3, respectively. TOP2B is the predominant form in quiescent and terminally differentiated cells, while TOP2A and TOP2B are both abundant in cycling cells [23,24]. 

Clinically relevant TOP2 poisons fall into several chemical classes including epipodophyllotoxins, acridines, anthracylines, the anthacenedione mitoxantrone and the benzo[c]phenanthridine alkaloid NK314 (which is also reported to down-regulate non homologous end-joining) [25,26,27] (Table 2). Antibacterial quinolones such as ciprofloxacin act through poisoning of the bacterial type II DNA topoisomerase (DNA gyrase and topoisomerase IV) and recently voreloxin, a quinolone derivative that targets human TOP2 was described [28]. 

**Table 2:** TOP2 poisons, those that are or have been used clinically in cancer therapy are in bold.

Chemical class	Examples
Epipodophyllotoxins	Etoposide, Teniposode
Anthracylines	Doxorubicin, Epirubicin, Daunorubicin, Idarubicin, Aclarubicin
Anthacenedione	Mitoxantrone
Acridines	m-AMSA (amsacrine), m-AMCA, AMCA, DACA
Quinalones	Voreloxin
Benzo[c]phenanthridine alkaloid	NK314

In addition a number of naturally occurring compounds including selenite, curcumin, digitoxin, dietary flavonoids and the green-tea constituent EGCG inhibit TOP2 or induce TOP2-DNA complexes in cell culture studies [29,30,31,32,33,34] and it has been suggested that flavonoids and catechins derived from the maternal diet may contribute to in utero *MLL* translocations underlying neonatal leukemias through their activity as topoisomerase poisons II [29,30,35,36]. As discussed below, *MLL* translocations found in neonatal acute leukemia cases share molecular features with those in t-AML.

The anthracyclines doxorubicin, daunorubicin and idarubicin and the anthracenedione, mitoxantrone differ from the epipodophyllotoxin TOP2 poisons (etoposide and teniposide) in that they are strong DNA intercalators. In addition to direct TOP2 poisoning and intercalation, ROS stimulated formaldehyde production, leads to the formation of anthracycline and mitoxantrone DNA adducts and crosslinks [37,38,39,40,41], which are presumably cytotoxic in their own right [42]. 

## 3. TOP2 Poisons and Chromosome Translocations

**Figure 4:**
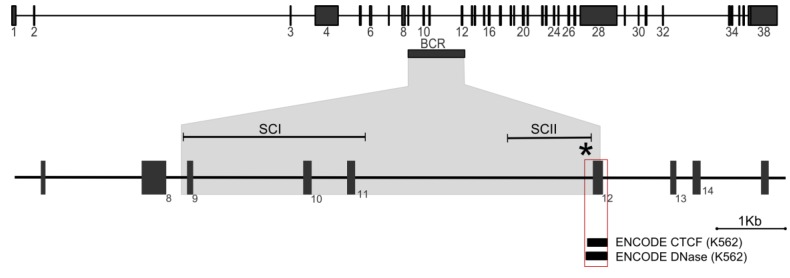
MLL translocations. Top: Genomic organization of the *MLL* gene, exons are numbered. Horizontal rectangle, breakpoint cluster region (BCR). Bottom: Enlarged segment of the *MLL* locus encompassing the breakpoint region. SCI and SCII denote BCR subclusters in which *de novo* AML and t-AL/neonatal translocation breakpoints are concentrated respectively [45]. The asterisk denotes a “hotspot” where nine tightly clustered therapy related or neonatal breakpoints have been reported. The red-boxed region highlights a peak of CTCF binding and DNase I hypersensitivity observed in the publically available ENCODE data (http://genome.ucsc.edu/cgi-bin/hgGatewa [56,57,58]).

TOP2 is a well validated anti-cancer target and TOP2 poisons are widely used and effective therapeutic agents; but, as discussed above, they are associated with the occurrence of late complications, including therapy-related acute leukemia. It is hoped that a better understanding of the events leading to t-AL, in particular the mechanism by which t-AL recurrent translocations occur may help minimize these side effects. Detailed analysis of translocation breakpoints (the position in a derivative chromosome where the two heterologous chromosome segments are fused) and aspects of TOP2 biology have begun to shed light on the mechanisms by which these translocations occur. For some of the genes involved, including *MLL, PML, RARA* and *AML1 (RUNX1)* a relatively large number of translocation breakpoints from *de novo* and therapy related leukemia cases have been determined at the base pair level. What has emerged from this is that while t-AL related breakpoints fall within previously identified breakpoint clusters (BCRs), their distribution can be skewed compared to the distribution of *de novo* AML breakpoints. This is particularly noticeable for the *MLL* gene. Chromosomal breakpoints involved in *MLL* translocations in acute leukemia fall within an 8-Kb breakpoint cluster region (BCR), but breakpoints reported from t-AL and neonatal acute leukemia cases (<1 year) are concentrated in the most telomeric 1 Kb of this region, while breakpoints from *de novo* cases cluster towards the centromeric end of this region (Figure 4) [43,44,45]. Similarly, for the *PML* gene, reported acute promyelocytic leukemia breakpoints fall into BCRs in introns three and six, but in leukemia cases associated with mitoxantrone treatment, there is a remarkably tight clustering of *PML* breakpoints within an 8 bp region of intron 6 [12,14]. This includes five cases of secondary leukemia following mitoxantrone treatment for multiple sclerosis [15] (see above). Returning to the *MLL* gene, the similar distribution of t-AL and neonatal acute lymphoblastic leukemia *MLL* translocation breakpoints [44] is intriguing since *MLL* translocations are particularly common in infant acute leukemia [46] and it has been suggested that TOP2 poisons acting *in utero* may play a role in the etiology of infant leukemias [30,35,44,47]. The telomeric end of the *MLL* BCR also contains an area of DNase I hypersensitivity [48,49], cryptic promoter activity and binding by the transcription factor CTCF (see Figure 4) [50]. DNase I hypersensitivity results from increased chromatin accessibility and is characteristic of promoters and other transcriptional regulatory elements. Intriguingly, amongst reported t-AML and neonatal acute leukemia *MLL* translocations that have been described at the DNA sequence level there is a breaksite hotspot [51,52,53] that coincides with the peak of DNase I hypersensitivity and CTCF binding (Figure 4). It has been suggested that an unusual chromatin structure and/or cryptic promoter activity contributes to *MLL* translocation in t-AL and possibly neonatal acute leukemia [49,50,54]. Furthermore, DNase I hypersensitive sites are also associated with BCRs of other genes that are rearranged in t-AML translocations including *AF9* and *AF4* (common *MLL* translocation partners), and in the *AML1*/*RUNX1* and *ETO* genes [48,55]. 

Recently, it has emerged that TOP2B has a role in transcription, being involved in the transcriptional response to estrogen and other nuclear hormones in a manner that appears to involve transient DSB induction [59,60,61,62,63]. In addition, inhibitor studies suggest that etoposide-induced TOP2B cleavage complexes are proteolytically processed to reveal DSBs in a transcription-linked mechanism [64]. Furthermore, TOP2 is also thought to relieve positive supercoiling ahead of elongating RNA polymerase [65] and to be required for efficient transcription on chromatin templates [21], although this process may involve TOP2A rather than TOP2B. DNase I hypersensitivity and cryptic promoter activity, together with the connections between TOP2B and transcription led us to suggest that DSBs introduced by TOP2B during transcription (and stabilized by a TOP2 poison) are the causative DSBs in chromosomal translocations in therapy related leukemia with anti-TOP2 drugs [66].

**Figure 5:**
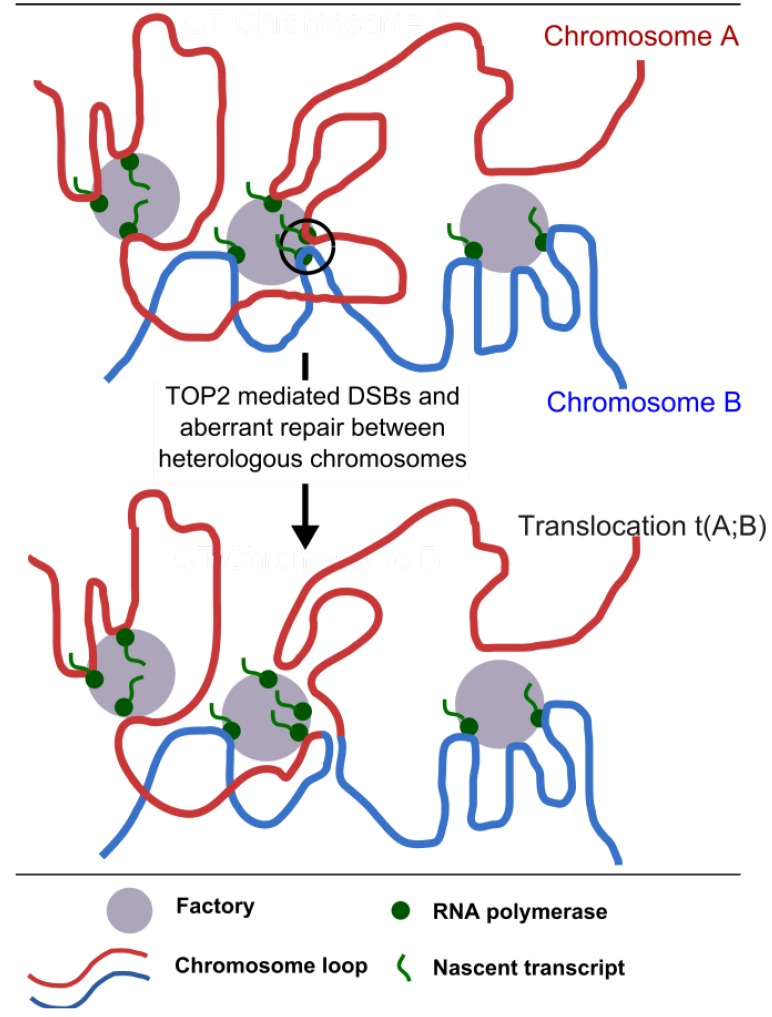
Clustering of transcription into transcription factories could facilitate chromosome translocation. Segments of two juxtaposed chromosomes are shown in red and blue. Transcription factories can contain active genes located on different chromosomes where these chromosomes come into contact and potentially intermingle [68]. Ongoing transcription in a shared transcription factory can maintain two heterologous chromosome segments in juxtaposition for the length of the transcription cycle (circled area, top part of the diagram) and it is hypothesized that this facilitates translocation when DNA breaks are induced. In the case of t-AML related translocations breaks are introduced by the action of TOP2 poisons.

When considering mechanisms that facilitate recurrent chromosome translocations, the processes that generate DSBs are only part of the story. For translocation to occur the participating genes must also be in the same nuclear vicinity to allow interaction and aberrant end-joining. Processes that bring these genes together or facilitate their interaction would be expected to increase the risk of a translocation between them. Interphase genomes are non-randomly arranged in the nuclear space and the average distance between genes appears to affect translocation frequency [67]. While the processes that organize the chromosomes in the interphase nucleus are not well understood, a contributing factor and one that could be relevant to chromosome translocations especially in relation to t-AML is the now well accepted concept of transcription factories (see Figure 5). 

Transcription occurs at dynamic structures called factories or hubs [69] consisting of multiple active RNA polymerase complexes each engaged with a transcription unit (gene) [70]. The dynamic association of genes with these focal sites of transcription results in looping and loops are presumed to form as transcription initiates, remain associated while transcription proceeds and then redistribute as transcription terminates (Figure 5). Since interphase chromosomes occupy distinct territories in the nucleus [68], transcription units derived from proximal regions of the same chromosome are likely to share transcription factories with the greatest frequency. However, high resolution FISH has demonstrated significant intermingling of chromatin between adjacent chromosome territories [68]. As a result genes residing on different chromosomes can simultaneously associate with common transcription factories, as has been demonstrated using RNA FISH to visualize sites of ongoing transcription [71,72]. In the context of hematological malignancies, the *Igh* and *Myc* genes (which are involved in translocations in B-cell lymphoma such that the proto-oncogene *Myc* is driven by the active *Igh* promoter) frequently share sites of transcription in stimulated mouse B cells [73]. Thus, sharing of transcription factories combined with transcription-associated TOP2-poison mediated DSBs could provide both the DSBs and the juxtaposition required for chromosome translocation (see Figure 5) in therapy related leukemias.

**Figure 6:**
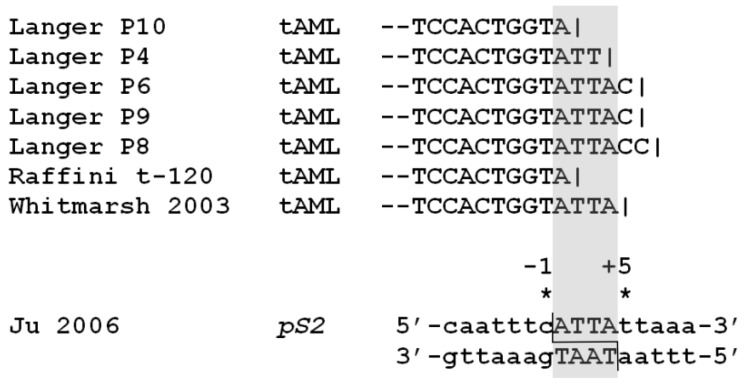
*MLL* translocation hotspot. *MLL* breakpoint sequences (der 11) from t-AL cases that form a “hotspot” near the telomeric end of the *MLL* BCR. The ATTA sequence is aligned with the same 4bp sequence identified in the etoposide-induced cleavage site described in the *pS2* promoter [59].

A key question concerning the etiology of the specific translocations observed in therapy related acute leukemia is why the *MLL* breakpoints occur in such a restricted genomic region in t-AL and neonatal acute leukemia compared to *de novo* AML (see Figure 4). This region is sensitive to etoposide-induced cleavage in cell line studies [49,50,74]; however, two distinct mechanisms have been implicated as sources of this sensitivity. The first is direct TOP2-mediated cleavage (as illustrated in Figure 3) and the second is related to early CAD-mediated apoptotic fragmentation [74,75,76]. The former model appears the simplest, and base-pair resolution mapping revealed etoposide-induced TOP2-mediated cleavage and DNA breaks in the SCII region of the *MLL* BCR, but notably it did not demonstrate preferential TOP2 mediated cleavage at the translocation hotspot referred to above [50,74] (Figure 4 and Figure 6). Higher order chromatin fragmentation occurring during TOP2 poison induced apoptosis was proposed as an alternative mechanism for initiation of t-AL–associated chromosomal translocations in cells that ultimately evade apoptosis and divide [74,75,76]. Base-pair resolution mapping revealed a major apoptotic cleavage region in *MLL* exon 12, however this is downstream of the BCR and more than 200 bp from the hotspot of *MLL* breaksites [74] (Figure 4 and Figure 6). Thus, the clustering of t-AML breakpoints cannot be explained simply by direct TOP2-mediated or apoptotic higher order cleavage patterns alone, but might result from interactions between these processes in conjunction with end-resection and other events associated with aberrant repair, or as discussed above, with transcription.

Interestingly, the hotspot of t-AL break sites described above is centered on an ATTA motif that is also found in the etoposide-stabilized TOP2B-associated cleavage site reported in the *pS2/TFF1* promoter (Figure 6) [59]. Although there is no strong consensus for optimum TOP2 cleavage sites [77], there is an apparent preference for A/T residues in the +1 to +4 positions which correspond to the 5’-overhang generated by TOP2 cleavage (Figure 6). TOP2 cleavage events at sites in two translocation partner genes could generate homologous 4 bp single-stranded overhangs (after cleavage complex processing, see below) that anneal to form junction sequences that are repaired by non-homologous end joining in an aberrant repair reaction (Figure 5). This type of reciprocal cleavage followed by aberrant joining between TAATTA sequences may account for a minority of *MLL-AF9* translocation events [52], but analysis of translocation junction sequences suggests that aberant joining typically involves resection to reveal microhomologies in the partner chromosomes [53,78]. It should be noted that the precise biochemical steps in the normal repair process through which TOP2 complexes are processed to yield DNA ends that can be ligated are not fully understood. For TOP2B, this process can include transcription dependent proteasomal degradation of covalently coupled TOP2B [64], which may be followed by additional “end polishing” steps. This type of processing would be compatible with direct aberrant joining of the resulting staggered ends or resection to reveal microhomologies prior to joining.

## 4. Conclusions and Possible Approaches to Reducing T-AML Associated Chromosome Translocations

TOP2 poisons are useful and effective anti-cancer drugs, but they are associated with therapy-related secondary leukemia, often bearing 11q23 translocations involving the *MLL* gene. The distribution of *MLL* chromosomal breakpoints in t-AL cases differs from that observed for *de novo* AML with clustering at the 3’-region of intron 11 associated with a region of DNase I hypersensitivity and CTCF binding (Figure 4 and Figure 6). Neither direct TOP2-mediated cleavage nor early apoptotic processes alone appear to explain the distribution of *MLL* break sites. Instead, interaction between these processes and/or with events downstream of initial DNA cleavage and other factors such as chromatin accessibility and ongoing transcription may influence t-AML translocation [48,49,50]. For drugs such as etoposide, TOP2A appears to be the most important of the two TOP2 isoforms with regard to induced cytotoxicity and therefore anti-cancer activity [26]. Despite this, we and others have found that etoposide-induced genotoxicity is largely dependent on TOP2B [30,66,79]. Recent data has revealed an important and unexpected role for TOP2B in transcription [59,60,61] and transcription dependent processing of TOP2B cleavage complexes to DSBs. The possibility that this is connected to the apparent genotoxic potential of TOP2B in the presence of TOP2 poisons provides an intriguing area for further study which will provide a better understanding of the events that trigger chromosome translocations in t-AL and could lead to strategies to minimize the occurrence of leukemia after treatment for a primary malignancy. One such strategy is specific TOP2 poison targeting of TOP2A, since genotoxic effects appear to be mostly mediated by TOP2B [66]. In fact, the TOP2 poisons shown in Table 1 are not identical in their relative activities against TOP2A and TOP2B and TOP2 poisons have been described that preferentially target one or the other isoform [26,80,81]. Similarly, if transcription-dependent, TOP2B mediated DSBs trigger t-AL translocations as suggested above, then harmful genotoxic effects may be minimized by employing anti-cancer regimens in which a transcriptional inhibitor precedes administration of a TOP2 poison. Notably transcription inhibitors are currently under investigation as potential anti-cancer agents in their own right [82]. As understanding of the molecular events leading to TOP2 poison associated t-AL improves we are optimistic that strategies can be identified that will minimize the occurrence of secondary effects such as therapy related leukemia following use of this extremely useful class of anti-cancer agent.
